# Noncoding RNA, friend or foe for nephrolithiasis?

**DOI:** 10.3389/fcell.2024.1457319

**Published:** 2024-11-20

**Authors:** Qing Wang, Zhenlu Yang, Xiaolong Chen, Yuanyuan Yang, Kehua Jiang

**Affiliations:** ^1^ Department of Urology, Guizhou Provincial People’s Hospital, Guiyang, Guizhou, China; ^2^ Department of Radiology, Guizhou Provincial People’s Hospital, Guiyang, Guizhou, China; ^3^ Department of Urology, Tongji Hospital, Tongji Medical College, Huazhong University of Science and Technology, Wuhan, Hubei, China

**Keywords:** ncRNAs, nephrolithiasis, calcium oxalate, biomarkers, therapeutic targets

## Abstract

Nephrolithiasis is one of the most common diseases in urology, characterized by notable incidence and recurrence rates, leading to significant morbidity and financial burden. Despite its prevalence, the precise mechanisms underlying stone formation remain incompletely understood, thus hindering significant advancements in kidney stone management over the past three decades. Investigating the pivotal biological molecules that govern stone formation has consistently been a challenging and high-priority task. A significant portion of mammalian genomes are transcribed into noncoding RNAs (ncRNAs), which have the ability to modulate gene expression and disease progression. They are thus emerging as a novel target class for diagnostics and pharmaceutical exploration. In recent years, the role of ncRNAs in stone formation has attracted burgeoning attention. They have been found to influence stone formation by regulating ion transportation, oxidative stress injury, inflammation, osteoblastic transformation, autophagy, and pyroptosis. These findings contributes new perspectives on the pathogenesis of nephrolithiasis. To enhance our understanding of the diagnostic and therapeutic potential of nephrolithiasis-associated ncRNAs, we summarized the expression profiles, biological functions, and clinical significance of these ncRNAs in the current review.

## Introduction

Nephrolithiasis is one of the most prevalent diseases in urology and often causes renal colic, urinary tract infections, and damage to renal function (Thongprayoon et al., 2020). It is estimated that 1%–15% of individuals may suffer from kidney stones during their lifetime (Romero et al., 2010). Global prevalence rates for nephrolithiasis vary from 1% to 20% (Skolarikos et al., 2022). In countries such as Sweden, Canada, and America, the prevalence is notably higher than 10% (Skolarikos et al., 2024). The prevalence of kidney stones is 6.4% in China and shows an increasing trend (Zeng et al., 2017). Despite significant advancements in surgical treatments for kidney stones, medical treatments and preventions have seen minimal changes over the past three decades. This is primarily due to unclear mechanisms underlying stone formation (Khan et al., 2021). The 10-year recurrence rate for kidney stones after surgical interventions is reported to be as high as 50%, resulting in a huge morbidity and financial burden (Khan et al., 2016). The cost of nephrolithiasis treatment in the USA was $2 billion in 2000 and is estimated to be $4.5 billion by 2030 (Antonelli et al., 2014). Thus, it is important to explore the pathogenesis of nephrolithiasis and provide a theoretical basis for its treatment and prevention.

Approximately 80% of kidney stones primarily consist of calcium oxalate (CaOx) crystals, intermixed with varying amounts of calcium phosphate (Khan et al., 2021). Hyperoxaluria and hypercalciuria stand as the most prevalent metabolic abnormalities among patients with nephrolithiasis (Letavernier and Daudon, 2018; Witting et al., 2021). They can trigger the precipitation of urinary crystals and increase the risk of stone formation. According to classical lithogenic theory, there are specific crystal-attachment sites within the renal collecting system, termed interstitial Randall’s plaque and intraluminal Randall’s plug (Khan et al., 2016). Early-formed tiny urinary crystals can adhere to these sites and aggregate, eventually growing into kidney stones. However, the pathogenesis of nephrolithiasis is not solely based on urine supersaturation, but also involves intricate interactions between lithogenic factors and cellular components (Khan et al., 2021). Biological responses induced by the lithogenic microenvironment, including oxidative stress, inflammation, osteogenic transformation, apoptosis, autophagy, pyroptosis, and ferroptosis, have a significant impact on the initiation and progression of kidney stones (Wang et al., 2021). Investigating key biological molecules which govern kidney stone formation remains a central focus and challenge.

While it has traditionally been assumed that genetic information is predominantly conveyed by proteins, recent findings indicate that the majority of mammalian genomes are transcribed into noncoding RNAs (ncRNAs) (Mattick and Makunin, 2006). Based on biological functions, ncRNAs are broadly classified into housekeeping and regulatory RNAs. Housekeeping ncRNAs include ribosomal RNAs (rRNAs), transfer RNAs (tRNAs), small nuclear RNAs (snRNAs), and small nucleolar RNAs (snoRNAs) (Hombach and Kretz, 2016). These ncRNAs are consistently expressed and contribute to essential cellular functions. Regulatory ncRNAs exhibit tissue- and disease-specific expression patterns. They can be roughly categorized into long ncRNAs (lncRNAs) with sizes greater than 200 nucleotides, and small ncRNAs less than 200 nucleotides, which include microRNAs (miRNAs), small interfering RNAs (siRNAs), circular RNAs (circRNAs), and piwi-associated RNAs (piRNAs) (Hombach and Kretz, 2016; Tomar et al., 2020). Functionally, ncRNAs can modulate gene expression and influence disease progression, therefore emerging as a novel class of targets for diagnostic and therapeutic interventions (Yang et al., 2021; Simonson and Das, 2015; Zhou et al., 2021).

An increasing number of studies have demonstrated that miRNAs, lncRNAs and circRNAs are closely associated with stone formation, offering potential biomarkers and therapeutic targets for nephrolithiasis. In the current review, we aimed to summarize the expression profiles, biological functions and mechanisms, and clinical significance of regulatory ncRNAs in kidney stone disease.

## Biogenesis and functions of miRNAs, lncRNAs, and circRNAs

MiRNAs are single-stranded molecules that are approximately 21–23 nucleotides in length (Lu and Rothenberg, 2018). In humans, the biogenesis of miRNAs involves sequential nuclear and cytoplasmic processing events. MiRNAs are first transcribed as long transcripts called pri-miRNAs in the nucleus (Lee et al., 2002). RNA pol II is thought to be responsible for pri-miRNA transcription. The nuclear cleavage of pri-miRNAs into pre-miRNAs is carried out by RNase III endonuclease Drosha and pasha (Hombach and Kretz, 2016). Then, pre-miRNAs are transported into the cytoplasm via the interaction between exportin-5 and Ran-GTP (Yi et al., 2003). The maturation of pre-miRNAs in the cytoplasm is carried out by RNase III endonuclease Dicer, which separates the loop structure and leaves imperfect double strands known as the miRNA: miRNA complex. Subsequently, double-stranded RNAs are incorporated into the RNA-induced silencing complex (RISC) (Vishnoi et al., 2017). Within this complex, mature single-stranded miRNAs bind to the Argonaute protein and act as a guide to partially complementary regions located in the 3′-untranslated region (UTR) of target mRNAs (Vishnoi et al., 2017). The subsequent binding of trinucleotide repeat containing adaptor 6 (TNRC6) proteins plays a pivotal role in the translational repression and degradation of target mRNAs (Hombach and Kretz, 2016). It is estimated that more than 60% of mRNAs contain miRNA target sites in their 3′-UTR. Furthermore, each miRNA is able to target several mRNAs, indicating a complex regulatory network between miRNAs and mRNAs (Sayed and Abdellatif, 2011). MiRNAs are involved in the pathogenesis of many diseases (Shi et al., 2021; Azhar et al., 2022; Juchnicka and Kuzmicki, 2021; Ho et al., 2022). Their potential value in diagnosis and treatment has attracted much attention. MiRNA inhibitors and mimics targeting various pathways are currently under development and undergoing evaluation in many clinical trials (Thakral and Ghoshal, 2015; Gomez et al., 2015).

LncRNAs are RNA molecules longer than 200 nucleotides that lack the potential to code for proteins. Compared to messenger RNAs (mRNAs), lncRNAs contain fewer and longer exons and are less evolutionarily conserved (Statello et al., 2021). The transcription and processing mechanisms of most lncRNAs are similar to those of mRNAs. Briefly, they are transcribed by RNA polymerase II, capped by 7-methyl guanosine (m7G) at their 5′ends, polyadenylated at their 3′ends, and exported to the cytoplasm by nuclear RNA export factor 1 (NXF1) (Statello et al., 2021). While a considerable portion of lncRNAs is retained in the nucleus, the exact underlying mechanisms remain largely elusive. The molecular functions of lncRNAs can be classified into signaling, decoying, scaffolding, and guiding (Wang and Chang, 2011). As signals, lncRNAs can act as transcription factors to regulate the expression of downstream genes in signaling pathways. As decoys, lncRNAs can bind to transcription factors and other chromatin proteins to disable them. Additionally, lncRNAs can sequester microRNA (miRNAs), preventing them from interacting with their target mRNAs. In the role of guides, lncRNAs can recruit chromatin-modifying enzymes to target genes. As scaffolds, lncRNAs bring proteins together to form ribonucleoprotein complexes, such as ribosomes. Implicated as key regulators in numerous pathophysiological processes including the cell cycle, cell proliferation, metabolism, and differentiation (Zhang et al., 2022; Xue et al., 2022; Chen M. et al., 2022), lncRNAs exhibit significant potential in the diagnosis and treatment of diseases (Chen Y. et al., 2022; Yu et al., 2020; Hussen et al., 2022; Winkle et al., 2021; Hashemi et al., 2022).

**FIGURE 1:**
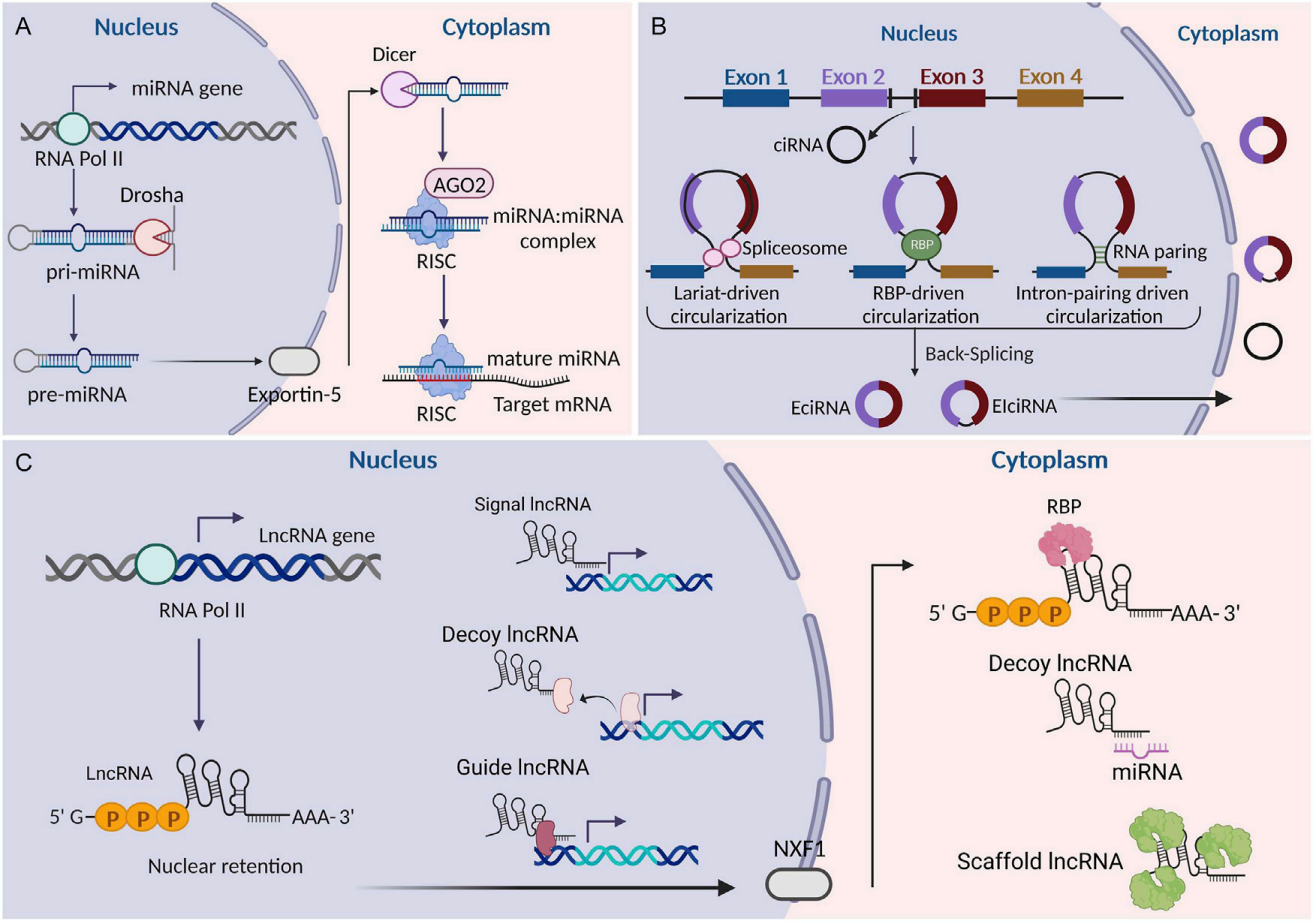
Biogenesis of miRNAs, lncRNAs, and circRNAs. **(A)** Biogenesis of miRNAs. **(B)** Biogenesis of circRNAs. **(C)** Biogenesis of lncRNAs. LncRNAs can be classified into signaling, decoying, scaffolding, and guiding lncRNAs according to their functions. EciRNA, exonic circRNA; CiRNA, circular intronic RNA; EIciRNA, exon-intron circRNA; RBP, RNA binding protein; AGO2, argonaute 2; NXF1, nuclear RNA export factor 1; RISC, RNA-induced silencing complex. This figure was created with BioRender.com.

As a novel type of ncRNA, circRNAs were first found in viroids in 1976 (Sanger et al., 1976) and were subsequently observed in the cytoplasm of eukaryotic cell lines in 1979 (Hsu and Coca-Prados, 1979). The most prominent feature of circRNAs is that they are covalently closed loops without 5′caps and 3′polyadenylated tails. This explains why circRNAs have a longer half-life and stronger exonuclease resistance than linear RNAs (Jeck and Sharpless, 2014). CircRNAs originate from the exons and/or introns of pre-mRNA transcripts by back splicing (Memczak et al., 2013). Circularization competes with canonical pre-mRNA splicing, with the production primarily governed by flanking intronic sequences (Ashwal-Fluss et al., 2014). CircRNAs were thought to lack open reading frames, leading to the assumption that they mainly functioned as miRNA sponges or RNA-protein complexes (Wilusz and Sharp, 2013; Kristensen et al., 2019). However, recent studies have identified that a small number of circRNAs can also be effectively translated into specific proteins (Xia et al., 2019; Pan et al., 2022). Owing to their remarkable stability, CircRNAs tend to accumulate in specific cell types. They show tissue-specific expression patterns and play crucial biological roles in various diseases, making them attractive potential candidates as diagnostic markers and therapeutic targets (Zhou et al., 2019; Mehta et al., 2020; Yang et al., 2019). Currently, circRNAs have become the focus of novel therapeutic strategies and vaccine development. Figure 1 illustrates the common biogenesis process of miRNAs, lncRNAs, and circRNAs.

## Integrated analysis of ncRNAs and mRNAs

Most ncRNAs do not code for proteins. Instead, they play important roles in mRNA regulation, chromatin organization, and other cellular processes. An integrated analysis of ncRNAs and mRNAs is a useful strategy for exploring the biological role of ncRNAs. Integrative data analysis involves pooling or combining two or more independent datasets into one, which is then statistically analyzed. Accordingly, integrated analysis of ncRNAs and mRNAs involves profiling the expression levels of both RNA types via high-throughput technologies (RNA sequencing or microarray analysis). It puts both types of RNAs together to gain a comprehensive understanding of gene expression regulation and its impact on cellular function and disease process (Kristensen et al., 2014). Common analyses include co-expression analysis, target prediction, functional enrichment analysis, and competing endogenous RNAs (ceRNA) network analysis (Zhang et al., 2019; Shvedova et al., 2016; Wang et al., 2023). These analyses reveal how ncRNAs regulate the expression of target mRNAs, modulate signaling pathways, and influence cellular processes. This information is essential and highly efficient for researchers in identifying intricate regulatory networks, novel biomarkers, and reliable therapeutic targets for various diseases.

## ncRNA expression profiles in kidney stone disease

**FIGURE 2:**
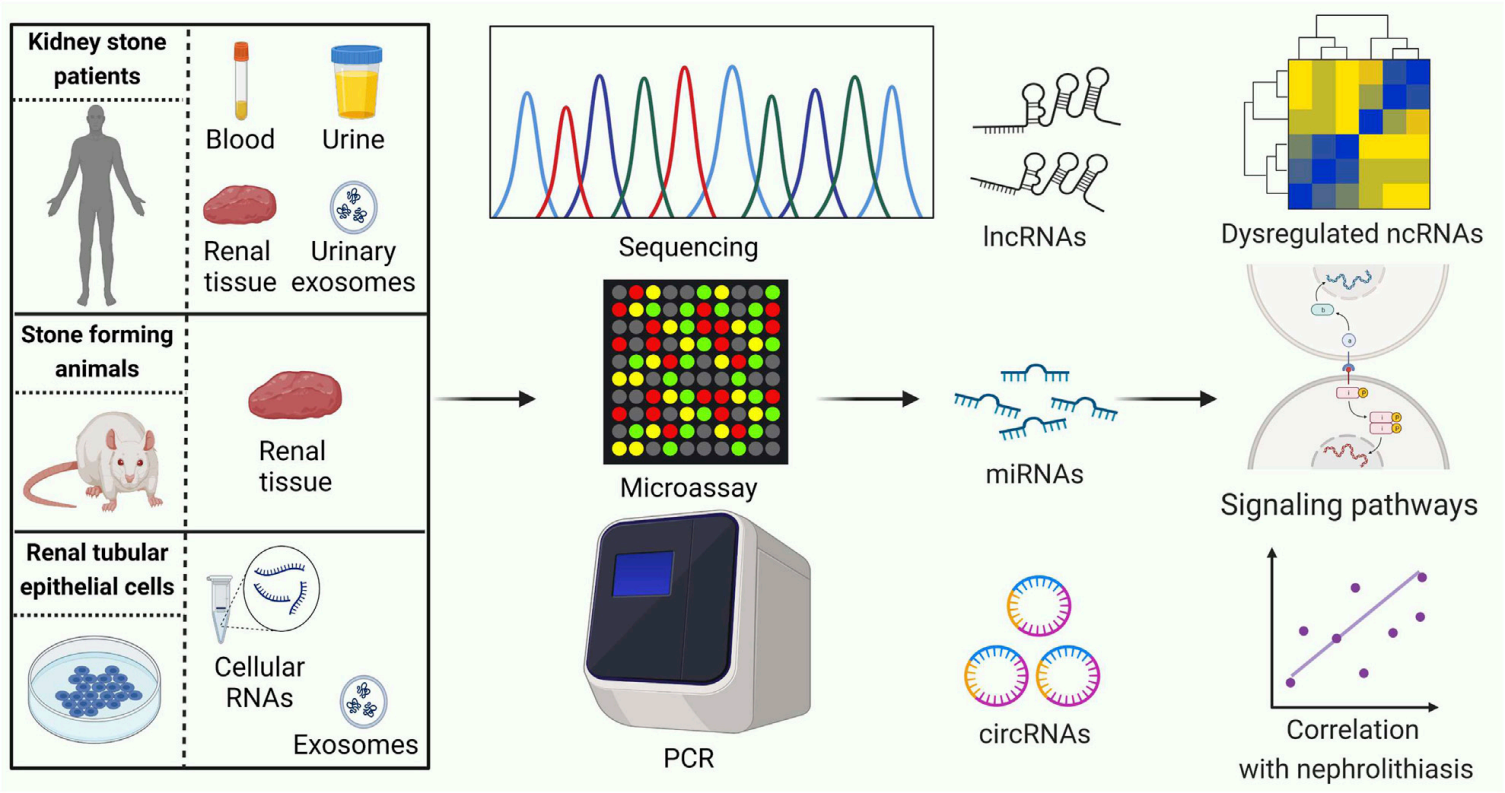
Current strategies for conducting ncRNA expression profiling studies related to kidney stones. PCR: polymerase chain reaction. LncRNAs, long noncoding RNAs; MiRNAs, microRNAs; CircRNAs, circular RNAs. This figure was created with BioRender.com.

Identifying ncRNAs with abnormal expression is an important strategy in the study of nephrolithiasis. In recent years, the rapid development of high-throughput genomics, especially microarray and RNA-sequencing technologies, has shown great advantages for exploring stone-related differential ncRNAs (Figure 2). An overview of ncRNAs identified by RNA sequencing and microarrays in kidney stone disease is listed in Table 1.

**TABLE 1:** Overview of ncRNAs identified by RNA sequencing and microarrays in kidney stone disease.

Year	Authors	Sample	Object	Sample size	Method	GEO ID	Dysregulated ncRNAs in lithogenic environment	ncRNAs validated by qRT-PCR
2014	Wang et al. (2014)	Cellular RNA	Control HK-2 cells	1	miRNAs Microarray	GSE56934	16 upregulated	4 upregulated (miR-638, miR-3125, miR-3195, miR-1260, miR-371- 5p)
			HK-2 cells treated with COM	1			9 downregulated	2 downregulated (miR-933, miR-4284)
2016	Lu et al. (2016)	Renal tissue	Healthy control rats	12	miRNAs Microarray	GSE75543	10 upregulated	4 upregulated (miR-184, miR-21-3p, miR-672-5p, miR-674-5p)
			GHS rats	12			9 downregulated	5 downregulated (miR-484, miR-138-1-3p, miR-201-3p, miR-203a-3p, miR-138-5p)
2016	Liu et al. (2016)	Renal tissue	Healthy control rats	3	miRNAs Microarray	GSE72135	10 upregulated	3 upregulated (miR-214-3p, miR-146b-5p, miR-31a-5p)
			CaOx nephrocalcinosis rats	3			9 downregulated	2 downregulated (miR-369-5p, miR-141-5p)
2017	Lan et al. (2017)	Renal tissue	Healthy control rats	8	miRNAs Microarray	NA	18 upregulated	4 upregulated (miR-132-3p, miR-181a-1-3p, miR-222-3p, miR-351-5p)
			CaOx nephrocalcinosis rats	8			20 downregulated	5 downregulated (miR-192-3p, miR-194-5p, miR-29c-3p, miR-185-5p, miR-30c-5p)
2018	Guan et al.	Cellular exosomes	Control HK-2 cells	3	RNA-seq	GSE110509	NA	NA
			HK-2 cells treated with oxalate	6				
2018	Zhang et al. (2017)	Renal tissue	Healthy control mice	3	lncRNAs Microarray	NA	154 upregulated lncRNAs	2 upregulated (AU015836, CHCHD4P4)
			CaOx nephrocalcinosis mice	3			222 downregulated lncRNAs	NA
2018	Cui et al.	Renal tissue	Normal human renal papillary tissue	3	lncRNAs Microarray	GSE117518	NA	NA
			Human renal papillary tissue with Randall’s plaques	3				
2018	Cao et al. (2018)	Renal tissue	Healthy control rats	4	RNA-seq	NA	711 upregulated lncRNAs	1 upregulated (TCONS_00030209)
							717 downregulated lncRNAs	2 downregulated (NONRATT009934.2, TCONS_00026280)
			CaOx nephrocalcinosis rats	4			58 upregulated circRNAs	NA
							87 downregulated circRNAs	NA
2019	Liang et al. (2019)	Urine	Healthy controls	6	miRNAs lncRNAs Microarray	NA	4 upregulated miRNAs	1 upregulated miRNA (miR-6796-3p)^a^
							5 downregulated miRNAs	4 downregulated miRNAs (miR-30d-5p, miR-3192–3p, miR-518b, miR-6776-3p)^a^
			CaOx kidney stone patients	5			790 upregulated lncRNAs	4 upregulated lncRNAs (lnc-FAM72B-4, lnc-EVI5L-1, lnc-SERPINI1-2, lnc-MB-6)^a^
							212 downregulated lncRNAs	2 upregulated lncRNAs (lnc-TIGD1L2–3, lnc-KIN-1)^a^
2022	Yang et al. (2022)	Urinary exosomes	Healthy controls	3	RNA-seq	NA	40 upregulated	NA
			CaOx kidney stone patients	3			14 downregulated	1 downregulated (miR-127-3p)
2020	Wang et al. (2019)	Cellular RNA	Control HK-2 cells	1	RNA-seq	NA	9 upregulated	9 upregulated (LINC00339, DHAP2, HCG18, RP11-381O7.3, LINC00115-201, PTPRG-AS1, MIR6516, RP11-504P24.3-005, LINC00665)
			HK-2 cells treated with COM	1			16 downregulated	16 downregulated (NIFK-AS1, RALY-AS1, LOC102724017, METTL8, THUMPD3-AS1 LINC00115, LINC00958, RP11-504P24.3, LINC00338-008, RP11-2C24.6-001, MIR22 RP11-566E18.3-201, CTB-60E11.4-001, AC005523.2-001, TFDP2-001, RP11-474G23.2-001)
2021	Xu et al.	Renal tissue	Healthy control mice	6	RNA-seq	GSE186793	NA	NA
			CaOx nephrocalcinosis mice	6				
2024	Zhu et al. (2024)	Urinary exosomes	Healthy controls	10	RNA-seq	NA	523 upregulated	miR-31-5p, miR-148b-5p, miR-205-5p, miR-574-5p
			CaOx kidney stone patients	10			296 downregulated	NA

NA, not applicable; CaOx, calcium oxalate; COM, calcium oxalate monohydrate; CircRNAs, circular RNAs; GEO, gene expression omnibus database; LncRNAs, long noncoding RNAs; MiRNAs, microRNAs; RNA-seq, RNA, sequencing.

^a^ Liang et al. validated the sequence results in HK-2, cells treated with oxalate.

Wang et al. (2014), Liang and Zhang used fold change>1.5 as the threshold, others used fold change>2 as the threshold.

Numerous studies have profiled ncRNA expression in renal tissue using stone-forming animal models, with a predominant focus on miRNAs. We investigated renal miRNA profiles using a microarray in 12 paired genetic hypercalciuric stone-forming (GHS) rats and normal Sprague-Dawley (SD) rats. Ten miRNAs were found to be upregulated and nine were downregulated (>2-fold change, *p* < 0.05) in GHS rats (Lu et al., 2016). Liu et al. (2016) and Lan et al. (2017) have both conducted microarray experiments to explore the renal expression of miRNAs in CaOx nephrocalcinosis rats. Liu et al. (2016) identified 20 upregulated and eight downregulated miRNAs (>2-fold change, *p* < 0.05) in nephrocalcinosis rats. Lan et al. (2017) identified 38 mature miRNAs. Among them, eighteen miRNAs were upregulated, while 20 were downregulated (>2-fold change, *p* < 0.05) in the stone group. Cao et al. (2018) explored lncRNA and circRNA expression profiles in the kidneys from CaOx nephrocalcinosis rats. As a result, the expression of 1,440 lncRNAs and 145 circRNAs was found to be altered (>2-fold change, *p* < 0.05 and false discovery rate (FDR) < 0.05). Additionally, Zhang et al. (2017) analyzed the lncRNA profiles in renal tissue from CaOx nephrocalcinosis mice and found that 154 lncRNAs were upregulated, while 222 lncRNAs were downregulated (>1.5-fold change, *p* < 0.05). Xu et al. also uploaded the renal miRNA-sequencing data from six paired normal C57BL/6J mice and CaOx nephrocalcinosis mice to the Gene Expression Omnibus (GEO) database (GSE186793).

ncRNA profiles in kidney stone patients have also been studied. Liang et al. (2019) investigated the urinary lncRNA and microRNA profiles using microarray technology in five CaOx stone patients and six healthy controls. Nine miRNAs and 1,002 lncRNAs were found to be dysregulated (>1.5-fold change, *p* < 0.05) in patients with nephrolithiasis, including four upregulated and five downregulated miRNAs and 790 upregulated and 212 downregulated lncRNAs. Yang et al. (2022) isolated urinary exosomes from the first morning voids of three paired CaOx stone patients and healthy controls. Urinary exosomal miRNA profiles were detected by sequencing. In the stone group, fourteen downregulated miRNAs and 40 upregulated miRNAs (>2-fold change, *p* < 0.01) were identified. Gene Ontology (GO) analysis and Kyoto Encyclopedia of Genes and Genomes (KEGG) pathway analysis indicated that these dysregulated miRNAs were enriched in oxidative stress, focal adhesion, cell adhesion molecule binding, and the mitogen-activated protein kinase signaling pathway. Similarly, Zhu et al. (2024) also detected the urinary exosomal miRNA profiles by sequencing in renal CaOx stone patients and found that 523 miRNAs were upregulated, while 296 miRNAs were downregulated (>2-fold change, *p* < 0.05). Cui et al. detected lncRNA profiles in human renal papillary tissues with Randall’s plaque (GSE117518).

*In vitro* studies are often conducted in human proximal renal tubular epithelial cells (HK-2). Wang et al. (2014) investigated miRNA profiles by microarray technology in HK-2 cells cultured with calcium oxalate monohydrate (COM). Twenty-five miRNAs were found to be differentially expressed with more than a 1.5-fold change. Among them, 16 were upregulated and nine were downregulated. Another sequencing study identified nine upregulated lncRNAs and 16 downregulated lncRNAs (>2-fold change, FDR<0.05) in HK-2 cells cultured with COM (Wang et al., 2019). Guan et al. have uploaded the miRNA-sequencing data of exosomes from COM-treated HK-2 cells to the GEO database (GSE110509).

## Biological functions and molecular mechanisms of ncRNAs in kidney stone disease

### Ion transportation

In addition to hypercalciuria and hyperoxaluria, other metabolic abnormalities, such as hyperuricosuria, hypomagnesuria, and hypocitraturia, can also increase the risk of nephrolithiasis (Skolarikos et al., 2024). Ameliorating the abnormal ion concentration in urine is a feasible method to prevent kidney stone formation. Oxalate is transferred from renal epithelial cells to the lumen through solute carrier family 26 member 6 (Slc26a6), while urinary citrate is mainly reabsorbed in the proximal renal tubule via solute carrier family 13 member 2 (Slc13a2). Lan et al. (2021) discovered that miR‐411‐3p directly bound to the 3′‐UTRs of both Slc26a6 and Slc13a2 mRNAs. Supplementation with glycine was found to upregulate miR-411-3p, leading to the inhibition of protein expression of Slc26a6 and Slc13a2 in renal tubular epithelial cells. This resulted in reduced urinary oxalate excretion and increased citrate excretion, ultimately leading to a decrease in renal CaOx crystal deposition. Zhu et al. (2019a) demonstrated that miR-130a-3p and miR-148b-3p could also target Slc13a2. Vinegar supplementation increased the expression of miR-130a-3p and miR-148b-3p in renal tubular epithelial cells. This led to the suppression of Slc13a2 expression and an enhancement in the concentration of urinary citric acid. Additionally, vinegar reduced urinary calcium excretion by stimulating miR-374b-5p gene transcription, thus suppressing the expression of Claudin-14, a potential treatment target for hypercalciuria. Zhu et al. (2024) further reported that miR-148b-5p could inhibit the expression of circRNA-83536, thereby reducing its suppression of miR-24-3p. MiR-24-3p could bind to the 3′‐UTRs of the calcitonin receptor (Calcr), then leading to downregulation of its expression. This cascade of events ultimately results in the promotion of urinary calcium excretion and stone formation. In a study by Cui et al. (2022), miR-103a-3p was found to negatively regulate the expression of transient receptor potential cation channel subfamily V member 5 (TRPV5) by targeting uromodulin. Silencing miR-103a-3p could activate the expression of TRPV5 and promote the reabsorption of calcium ions by renal tubular epithelial cells. This lead to a reduction in urinary calcium excretion and crystal deposition in the kidneys of hyperoxaluria rats.

### Oxidative stress injury

Stone formation is closely associated with an increase in reactive oxygen species (ROS) within the kidney (Khan, 2014). ROS generated during aerobic metabolism encompass diverse molecules such as superoxide anion, nitric oxide free radical, hydroxyl, hydrogen peroxide, and so on (Yang and Lian, 2020). In general, the production and clearance of cellular ROS are balanced to maintain physiological functions. However, under certain pathological circumstances, such as stimulation by calcium oxalate (CaOx) crystals or elevated concentrations of oxalate or calcium, the production of ROS escalates while clearance mechanisms become insufficient. This imbalance leads to oxidative stress injury and then promotes the adhesion of crystals to renal tubular epithelial cells (Chaiyarit and Thongboonkerd, 2020; Albert et al., 2020).

SIRT1 is an NAD-dependent protein deacetylase showing anti-inflammatory and antioxidative functions. Ye et al. (2021) found that miR-128-3p was upregulated in the kidneys of CaOx nephrocalcinosis mice and it suppressed the expression of SIRT1 by binding its 3′-UTR. Theaflavin supplementation could alleviate CaOx-induced renal oxidative stress injury by inhibiting miR-128-3p while upregulating SIRT1. In our previous study, we demonstrated that the expression of miR-155-5p was increased in HK-2 cells cocultured with COM and that miR-155-5p could directly target matrix Gla protein (MGP). Additionally, miR-155-5p antagonist significantly reduced renal crystal disposition and ROS generation in CaOx nephrocalcinosis rats (Jiang et al., 2020). Wang X. et al. (2020a) reported that the expression of miR-30c-5p expression was significantly downregulated in HK-2 cells treated with sodium oxalate. Overexpression of miR-30c-5p reversed apoptosis, crystal deposition, lactate dehydrogenase, malondialdehyde and ROS levels in the kidneys of CaOx nephrocalcinosis mice by targeting autophagy protein 5 (ATG5). In addition, another study has shown that supplementation with miR-204 agonists could reduce CaOx-induced oxidative stress injury in renal tubular epithelial cells, offering potential targets for preventing stone formation (Xie et al., 2022).

It has been reported that NACHT, LRR and PYD domain-containing protein 3 (NLRP3) plays a crucial role in inflammation. Lv et al. found that miR-223-3p bound to the 3′-UTR of both NLRP3 and lncRNA X inactive specific transcript (XIST). CaOx could upregulate the expression of XIST, which competitively bound to miR-223-3p, leading to the release of NLRP3 mRNA from miR-223-3p in renal tubular epithelial cells. These competing interactions between XIST/miRNA-223/NLRP3 ultimately exacerbated the production of ROS, inflammatory injury, and crystal deposition in the renal tissue of CaOx nephrocalcinosis mice (Lv et al., 2021). Similarly, studies regarding XIST sponging miRNAs to activate NLRP3 inflammasome in other diseases have also been reported (Ma et al., 2019; Liu et al., 2021; Zhao et al., 2022). Evidence has shown that lncRNA H19 is involved in inflammatory regulation and induces tissue injury (Song et al., 2017; Wang et al., 2017). Genome-wide expression profile analysis of renal tissue with Randall’s plaques demonstrated that H19 expression was upregulated (Taguchi et al., 2017). Liu et al. (2019) found that miR-216b bound to the 3′-UTR of both H19 and high mobility group protein B1 (HMGB1). H19 could activate the HMGB1/TLR4/NF-κB pathway by competitively binding miR-216b in the kidney. This activation subsequently induced oxidative stress injury and promoted stone formation. Li et al. (2021) reported that the expression of lncRNA ATB was increased, while the expression of miR-200a was decreased in HK-2 cells after incubation with COM. Further investigation revealed that miR-200a directly bound to the 3′-UTR of lncRNA ATB, as confirmed by a dual-luciferase reporter experiment. ATB promoted ROS generation, epithelial-mesenchymal transition, and apoptosis to contribute to CaOx-induced renal injury by sponging miR-200a. It should be indicated that competing regulations between ATB and miR-200 family also played a role in the pathogenesis of carcinoma and neurotoxicity (Wang et al., 2018; Li et al., 2018; Zhu L. et al., 2020). Li et al. (2022) found that the expression of miR-484 was downregulated in the renal tissue of CaOx nephrocalcinosis rats. MiR-484 could upregulate the expression of forkhead box protein O1 (FoxO1) by targeting Vitamin D receptor (VDR), then suppressing apoptosis and oxidative stress injury in renal tubular epithelial cells. Han et al. (2023) reported that miR-184 was upregulated in both the renal tissue of CaOx nephrocalcinosis rats and renal tubular epithelial cells (HK-2) cultured with COM. This miRNA is capable of directly binding to the 3′-UTR of insulin-like growth factor 1 receptor (IGF1R), subsequently promoting COM-induced oxidative stress injury and apoptosis in renal tubular epithelial cells.

### Inflammation

In recent years, increasing attention has been given to the role of the immune response in kidney stone formation (Deng et al., 2018). The lithogenic microenvironment can continuously stimulate renal tubular epithelial cells to secrete inflammatory factors. This results in an amplified inflammatory response that leads to renal tissue damage, promoting adhesion, aggregation, and growth of urine crystals. Moreover, macrophages are significantly increased in the kidneys of patients with CaOx nephrolithiasis, indicating their involvement in the immune regulation (Taguchi et al., 2021; Kumar et al., 2022). Studies have shown that M1 macrophages secrete proinflammatory factors and promote kidney stone formation, whereas M2 macrophages secrete anti-inflammatory factors and play a role in inhibiting stone formation (Khan et al., 2021; Dominguez-Gutierrez et al., 2020).

Li et al. (2020) reported that lncRNA HOXA11‐AS was upregulated in the renal tissue of CaOx nephrocalcinosis mice, while miR‐124‐3p was downregulated. It was observed that miR‐124‐3p had the ability to bind to the 3′-UTR of both monocyte chemotactic protein 1 (MCP-1) and HOXA11‐AS. LncRNA HOXA11‐AS acted as a competing endogenous RNA to promote apoptosis, aggravate cellular damage and upregulate MCP‐1 expression by sponging miR‐124‐3p, thereby mediating CaOx crystal-related renal inflammation. Macrophage colony-stimulating factor 1 (CSF-1) can recruit macrophages and promote M2 polarization. Zhu et al. (2019b) found that androgen receptor upregulated miR-185-5p by binding to the androgen response element on its 5ʹ promoter in renal tubular epithelial cells. Dual luciferase reporter experiments showed that miR-185-5p bound to the 3′-UTR of CSF-1. Silencing the androgen receptor (AR) in renal tubular epithelial cells lead to upregulation of CSF-1 expression by downregulating miR-185-5p. This ultimately promoted macrophage recruitment and M2 polarization, which reduced renal crystal disposition in CaOx nephrocalcinosis rats.

The interferon regulatory factor (IRF) family is a class of important nuclear transcription factors that regulate the polarization of macrophages. The expression of IRF-1 is increased in the kidneys of mice with hyperoxaluria, and IRF-1 is considered to play a lithogenic role. Liu et al. (2020) found that nuclear factor erythroid 2-related 2 (Nrf2) exhibited positive transcriptional activation of miR-93-5p by binding to its promoter. MiR-93-5p could target IRF1 and Toll-like receptor 4 (TLR4) mRNAs to inhibit their expression in macrophages. Sulforaphane, a pharmacological activator of Nrf2, could promote M2 macrophage polarization to suppress renal inflammation and crystal adhesion in CaOx nephrocalcinosis mice. Pioglitazone has also been reported to negatively regulate IRF1 expression by upregulating miR-23 in macrophages and then decreasing CaOx nephrocalcinosis (Chen et al., 2019). In another study by Yang et al. (2020), bioinformatics analysis and chromatin immunoprecipitation assays confirmed that the aryl hydrocarbon receptor (AhR) in bone marrow-derived macrophages could bind the promoter to transcriptionally activate miR-142a-3p. This activation subsequently inhibited the expression of IRF1 and HIF-1α by directly targeting their 3′-UTRs. AhR activation could diminish M1 macrophage polarization and promote M2 polarization to suppress CaOx nephrocalcinosis via the miR-142a-IRF1/HIF-1α pathway. In addition, Zhang et al. (2023) reported that adipose-derived stem cells (ADSCs)-derived miR-23-enriched exosomes could inhibit the polarization of M1 macrophages and protects against CaOx stone formation by directly targeting IRF1 in macrophages. Zhu et al. (2023) found that miR-493-3p bound to the 3′-UTR of the macrophage migration inhibitory factor (MIF). Acetate supplementation could enhance the H3K9 and H3K27 acetylation levels at the promoter region of miR-493-3p, subsequently upregulating miR-493-3p and downregulating MIF expression. This process decreased macrophage infiltration, therefore attenuating hyperoxaluria-induced renal injury.

### Osteogenic transformation

Randall’s plaque is an ectopic calcification located in the renal papillary interstitium. Its main component is hydroxyapatite, which is similar to the bone matrix (Wiener et al., 2018). The plaque can gradually grow into the renal interstitium and eventually break through the urothelium and contact urine. Early-formed urinary crystals can adhere to the plaque and then form stones. Therefore, Randall’s plaque is considered to be the origin of kidney stones by many researchers (Daudon et al., 2015). It has been reported that osteogenic transformation of cells in the renal collecting system, including renal tubular epithelial cells and renal interstitial fibroblasts, plays a role in the formation of Randall’s plaque (Gambaro et al., 2004; Joshi et al., 2015; Jia et al., 2014).

Cui et al. (2020) identified a series of ncRNAs that regulated the osteogenic transformation of renal interstitial fibroblasts in a lithogenic microenvironment. They found that the expression of miR-410-3p was decreased in renal interstitial fibroblasts cultured with osteoblastic medium and in renal papillary tissue with Randall’s plaque. A binding relationship between homeobox protein MSX-2 (MSX2) and miR-410-3p was confirmed through dual-luciferase reporter gene analysis. miR-410-3p could target MSX-2 to inhibit the osteogenic transformation of renal interstitial fibroblasts. Bone morphogenetic protein 2 (BMP2) has been reported to be a classical phenotypic molecule during osteogenic transformation. It has been reported that lncRNA NEAT1 may act as a key activator of BMP2, thus promoting the osteogenic differentiation of renal interstitial fibroblasts. In the cytoplasm, NEAT1 sponges miR-129-5p to stabilize BMP2 mRNA. In the nucleus, NEAT1 induces the nucleolar translocation of early growth response protein 1 (EGR1), which then binds to the promotor of BMP2 and promotes its transcription (Zhu et al., 2021). The expression of LncRNA MALAT1 was also found to be increased in human renal papillary tissue with Randall’s plaque. miR-320a-5p could bind to the 3′-UTR of both MALAT1 and runt-related transcription factor 2 (Runx2). MALAT1 acted as a sponge of miR-320a-5p to promote Runx2 expression, thus upregulating the expression of osteogenesis-associated proteins in renal interstitial fibroblasts (Zhu Z. et al., 2020).

### Other roles

Hu et al. (2014) compared the serum and urinary levels of miR-155 between 60 patients with nephrolithiasis and 50 healthy volunteers. They found that both serum and urinary levels of miR-155 were significantly higher in nephrolithiasis patients. They observed a positive correlation between plasma C-reactive protein (CRP) levels and urinary miR-155 levels. Additionally, the urinary expression of IL-1β, IL-6, and TNF-α showed an inverse correlation with urinary miR-155 levels. Chen et al. (2020) explored the exact role of miR-155 in stone formation. Similarly, they found that serum and renal levels of miR-155 were elevated in patients with CaOx stones. PI3K and Rheb were identified as downstream targets of miR-155. They ultimately showed that miR-155 facilitated CaOx crystal-induced renal tubular epithelial cell injury through PI3K-mediated autophagy. Shi et al. (2019) reported that miR‐20b‐3p was decreased in the renal tissue of CaOx nephrocalcinosis rats and in the urine of CaOx stone patients. Treatment with miR‐20b‐3p‐enriched exosomes protected renal function in CaOx nephrocalcinosis rats. *In vitro* experiments confirmed that miR‐20b‐3p‐enriched exosomes could alleviate oxalate‐induced autophagy and crystal adhesions in renal tubular epithelial cells by targeting autophagy protein 7 (ATG7) and TLR4. Gan et al. (2022) and Song et al. (2019) reported that CaOx crystals could suppress the expression of miR-22-3p and miR-141-3p in renal tubular epithelial cells, leading to the activation of NLRP3-mediated pyroptosis. This, in turn, promoted cell injury and crystal adhesion. In addition, the lncRNA LINC00339 was upregulated in HK-2 cells treated with COM and could sponge miR‐22‐3p (Song et al., 2019). Su et al. (2020) reported that miR-21 was elevated in the renal tissue and urine of patients with CaOx stones. miR-21 promoted CaOx-induced apoptosis and lipid accumulation in renal tubular cells by targeting peroxisome proliferator-activated receptor alpha (PPARA). Wang B. et al. (2020b) demonstrated that miR-34a could inhibit the expression of the adhesion molecule CD44 to reduce COM adhesions to renal tubular epithelial cells. The expression of LncRNA CHCHD4P4 was upregulated in the renal tissue of CaOx nephrocalcinosis mice. This change promoted epithelial-mesenchymal transition in CaOx-induced renal damage (Zhang et al., 2017). Xi et al. (2023) demonstrated that lncRNA LINC01197 played an inhibitory role in stone formation by sponging miR-516b-5p and subsequently activating the SIRT3/FOXO1 signaling pathway. An overview of dysregulated ncRNAs in kidney stone disease is shown in Table 2.

**TABLE 2:** Dysregulated ncRNAs in kidney stone disease.

Year	Authors	Name	Expression	Target gene	Specific cell lines	Function
2014	Hu et al. (2014)	miR-155	Upregulated in serum and urine from patients with nephrolithiasis	NA	NA	miR-155 may play a role in the pathophysiology of nephrolithiasis via regulating inflammatory cytokines expression
2018	Zhang et al. (2017)	LncRNA CHCHD4P4	Upregulated in renal tubular epithelial cells (HK-2) cultured with COM and renal tissue of CaOx nephrocalcinosis mice	NA	Renal tubular epithelial cells	CHCHD4P4 promotes epithelial-mesenchymal transition and inhibits cell proliferation in calcium oxalate-induced kidney damage
2019	Shi et al. (2019)	miR-20b-3p	Downregulated in renal tubular epithelial cells (HK-2) cultured with oxalate, the renal tissue of CaOx nephrocalcinosis rats, and the urine of CaOx stone patients	ATG7, TLR4	Renal tubular epithelial cells	Exosomes from miR-20b-3p-overexpressing ADSCs can protect renal function in CaOx nephrocalcinosis rats by suppressing autophagy and inflammatory responses
2019	Chen et al. (2019)	miR-23	Not change in renal tubular epithelial cells cultured with COM	IRF1, PKNOX1	Macrophages	Upregulating miR-23 via pioglitazone supplementation can diminish M1 macrophage polarization to suppress CaOx nephrocalcinosis
2019	Zhu et al. (2019a)	miR-130a-3p, miR-148b-3p, miR-374b-5p	Downregulated in renal tubular epithelial cells (HK-2 and NRK52E) cultured with oxalate and the renal tissue of CaOx nephrocalcinosis rats	Slc13a2, CLDN14	Renal tubular epithelial cells	Upregulating miR-130a-3p, miR-148b-3p, and miR-374b-5p via vinegar supplementation can increase the citrate excretion and reduce the calcium excretion to suppress CaOx nephrocalcinosis
2019	Song et al. (2019)	LncRNA LINC00339	Upregulated in renal tubular epithelial cells (HK-2) cultured with COM	miR-22-3p	Renal tubular epithelial cells	LncRNA LINC00339 promotes pyroptosis in renal tubular epithelial cocultured with COM by sponging miR‐22‐3p and activating the NLRP3 pathway
		miR-22-3p	Downregulated in renal tubular epithelial cells (HK-2) cultured with COM	LINC00339, NLRP3		
2019	Zhu et al. (2019b)	miR-185-5p	Downregulated in renal tubular epithelial cells knocked down androgen receptor, renal tissue of knocked down androgen receptor mice	CSF-1	Renal tubular epithelial cells	miR-185-5p can promote M2 macrophage polarization to suppress CaOx nephrocalcinosis
2019	Liu et al. (2019)	LncRNA H19	Upregulated in the renal tissue of murine CaOx nephrocalcinosis model	miR-216b	Renal tubular epithelial cells	LncRNA H19 activates the HMGB1/TLR4/NF-kB pathway by competitively binding miR-216b in kidney, which then induces oxidative stress injury and promotes stone formation
		miR-216b	Downregulated in the renal tissue of murine CaOx nephrocalcinosis model	H19, HMGB-1		
2020	Yang et al. (2020)	miR-142a-3p	NA	IRF1, HIF-1α	Macrophages	Upregulating miR-142a-3p aryl hydrocarbon receptor activation can diminish M1 macrophage polarization and promote M2 polarization to suppress CaOx nephrocalcinosis
2020	Jiang et al. (2020)	miR-155-5p	Upregulated in renal tubular epithelial cells (HK-2) cultured with oxalate or COM	MGP	Renal tubular epithelial cells	miR-155-5p promotes oxalate- and COM-induced kidney oxidative stress injury and CaOx nephrocalcinosis by suppressing MGP expression
2020	Su et al. (2020)	miR-21	Upregulated in renal tubular epithelial cells (HK-2) cultured with COM, renal tissue of CaOx nephrocalcinosis rats, renal tissue and urine of patients with CaOx stones	PPARA	Renal tubular epithelial cells	miR-21 promoted CaOx-induced apoptosis and lipid accumulation in renal tubular cells by targeting PPARA.
2020	Liu et al. (2020)	miR-93-5p	NA	IRF1, TLR4	Macrophages	Upregulating miR-93-5p by sulforaphane supplementation can diminish M1 macrophage polarization to suppress renal inflammatory injury and crystal deposition in CaOx nephrocalcinosis
2020	Wang et al. (2020a)	miR-30c-5p	Downregulated in renal tubular epithelial cells (HK-2) cultured with oxalate	ATG5	Renal tubular epithelial cells	miR-30c-5p reduces oxidative stress injury in renal tubular epithelial cells and inhibits the crystal adhesion
2020	Chen et al. (2020)	miR-155	Upregulated in renal tubular epithelial cells (HK-2) cultured with COM and the renal tissue and serum of patients with CaOx stones	PI3K, Rheb	Renal tubular epithelial cells	miR-155 facilitates CaOx crystal-induced renal tubular epithelial cells injury via targeting PI3K associated autophagy
2020	Cui et al., (2020)	miR-410-3p	Downregulated in renal tissue with Randall’s plaque and human renal interstitial fibroblasts cultured with calcium chloride	MSX2	Renal interstitial fibroblasts	miR-410-3p suppresses osteogenic differentiation of human renal interstitial fibroblasts cultured with calcium chloride through MSX2
2020	Wang et al. (2020b)	miR-34a	Downregulated in renal tubular epithelial cells (HK-2) cultured with COM	CD44	Renal tubular epithelial cells	miRNA-34a inhibits CaOx crystal adhesion by targeting CD44 in renal epithelial cells
2020	Li et al. (2020)	LncRNA HOXA11‐AS	Upregulated in renal tubular epithelial cells cultured with COM, and the renal tissue of CaOx nephrocalcinosis mice	miR-124-3p	Renal tubular epithelial cells	HOXA11‐AS functions as a completing endogenous RNA to promote apoptosis and aggravate cellular damage and upregulate MCP‐1 expression through sponging miR‐124‐3p, thereby mediating CaOx crystal-related renal inflammation
		miR-124-3p	Downregulated in renal tubular epithelial cells cultured with COM, and the renal tissue of CaOx nephrocalcinosis mice	HOXA11‐AS, MCP-1		
2021	Ye et al. (2021)	miR-128-3p	Upregulated in the renal tissue of CaOx nephrocalcinosis mice	SIRT1	Renal tubular epithelial cells	Downregulating miR-128-3p via theaflavin supplementation can suppress CaOx-induced kidney oxidative stress injury and CaOx nephrocalcinosis
2021	Lv et al. (2021)	LncRNA XIST	Upregulated in renal tubular epithelial cells (HK-2) cultured with COM and the renal tissue of CaOx nephrocalcinosis mice	miR-223-3p	Renal tubular epithelial cells	lncRNA XIST participates in the formation and progression of renal calculus by interacting with miR-223-3p and the NLRP3/Caspase-1/IL-1β pathway to mediate the inflammatory response and ROS production
		miR-223-3p	Downregulated in renal tubular epithelial cells (HK-2) cultured with COM	XIST, NLRP3		
2021	Lan et al. (2021)	miR‐411‐3p	Downregulated in renal tubular epithelial cells (HK-2 and NRK52E) cultured with oxalate and the renal tissue of CaOx nephrocalcinosis rats	Slc26a6, Slc13a2	Renal tubular epithelial cells	Upregulating miR‐411‐3p via glycine supplementation can reduce the urinary oxalate excretion and increase the citrate excretion to suppress CaOx nephrocalcinosis
2021	Li et al. (2021)	LncRNA ATB	Upregulated in renal tubular epithelial cells (HK-2) cultured with COM	miR-200a	Renal tubular epithelial cells	LncRNA ATB promotes the ROS generation, epithelial-mesenchymal transition, and apoptosis to participate in the process of CaOx-induced renal injury by sponging miR-200a
		miR-200a	Downregulated in renal tubular epithelial cells (HK-2) cultured with COM	ATB		
2021	Zhu et al. (2021)	LncRNA NEAT1	Upregulated in renal tissue with Randall’s plaque and human renal interstitial fibroblasts cultured with osteogenic medium	miR-129-5p	Renal interstitial fibroblasts	NEAT1 functioned as a key mediator of BMP2 to promote the osteogenic differentiation of human renal interstitial fibroblasts by inducing the nucleolar translocation of EGR1 and sponging miR-129-5p
		miR-129-5p	Downregulated in human renal interstitial fibroblasts cultured with osteogenic medium	NEAT1, BMP2		
2021	Zhu et al. (2020b)	LncRNA MALAT1	Upregulated in renal tissue with Randall’s plaque and human renal interstitial fibroblasts cultured with osteogenic medium	miR-320a-5p	Renal interstitial fibroblasts	MALAT1 promotes Runx2 expression to regulate osteogenic differentiation of human renal interstitial fibroblasts by sponging miRNA-320a-5p
		miR-320a-5p	NA	MALAT1, Runx2		
2022	Cui et al. (2022)	miR-103a-3p	NA	UMOD	Renal tubular epithelial cells	miR-103a-3p silencing ameliorates calcium oxalate deposition by activating the UMOD/TRPV5 axis to reduce urinary calcium excretion
2022	Xie et al. (2022)	miR-204	NA	MUC4	Renal tubular epithelial cells	miR-204 suppresses the renal oxidative stress injury and CaOx nephrocalcinosis via inhibiting MUC4/ERK signaling axis
2022	Gan et al. (2022)	miR-141-3p	Downregulated in renal tubular epithelial cells (HK-2) cultured with COM	NLRP3	Renal tubular epithelial cells	miR-141-3p represses pyroptosis by suppressing NLRP3 expression, thus protecting CaOx crystal-induced renal tubular epithelial cells injury
2022	Li et al., (2022)	miR-484	Downregulated in the renal tissue of CaOx nephrocalcinosis rats	VDR	Renal tubular epithelial cells	miR-484 suppresses apoptosis and prevents attachment of calcium oxalate crystals to renal tubular epithelial cells by inhibiting the VDR and subsequently upregulating the FoxO1
2023	Han et al. (2023)	miR-184	Upregulated in the renal tissue of CaOx nephrocalcinosis rats and renal tubular epithelial cells (HK-2) cultured with COM	IGF1R	Renal tubular epithelial cells	miR-184 promotes apoptosis and oxidative stress injury in renal tubular epithelial cells by targeting IGF1R
2023	Xi et al. (2023)	LINC01197	Downregulated in the renal tissue of patients with renal CaOx stones and renal tubular epithelial cells (HK-2) cultured with COM	miR-516b-5p	Renal tubular epithelial cells	LINC01197 inhibites the formation of kidney stones by regulating miR-516b-5p/SIRT3/FOXO1 signaling pathway
2023	Zhang et al. (2023)	miR-23	NA	IRF1	Macrophages	Adipose-derived stem cells-derived miR-23-enriched exosomes inhibites the polarization of M1 macrophages and protects against CaOx stone formation by directly targeting IRF1
2023	Zhu et al. (2023)	miR-493-3p	Downregulated in the renal tissue of CaOx nephrocalcinosis rats and renal tubular epithelial cells (HK-2) cultured with oxalate	MIF	Renal tubular epithelial cells	Upregulating miR-493-3p via acetate supplementation can downregulate MIF expression and decrease macrophages infiltration to attenuate the hyperoxaluria-induced renal injury
2024	Zhu et al. (2024)	miR-148b-5p	Upregulated in urinary exosomes derived from patients with renal CaOx stones	circRNA-83536	Renal tubular epithelial cells	miR148b-5p enhances urinary calcium excretion and contributes to the formation of calcium-containing kidney stones via the circRNA-83536/miR-24-3p/Calcr signaling pathway

ATG5, autophagy protein 5; ATG7, autophagy protein 7; BMP2, bone morphogenetic protein 2; CaOx, calcium oxalate; CLDN14, claudin-14; COM, calcium oxalate monohydrate; CSF-1, macrophage colony-stimulating factor 1; HIF-1α, Hypoxia-inducible factor 1-α; HMGB1, high mobility group protein B1; IRF1, interferon regulatory factor-1; MCP-1, monocyte chemotactic protein 1; MGP, matrix Gla protein; MSX2, homeobox protein MSX-2; MUC4, mucin-4; Slc13a2, solute carrier family 13 member 2; NLRP3, NACHT, LRR, and PYD, domain-containing protein 3; PI3K, Phosphatidylinositol-3-hydroxykinase; PPARA, peroxisome proliferator-activated receptor alpha; Rheb, GTP-binding protein Rheb; Runx2, runt-related transcription factor 2; SIRT1, NAD-dependent protein deacetylase sirtuin-1; Slc26a6, solute carrier family 26 member 6; TLR4, toll-like receptor 4; UMOD, uromodulin; VDR, vitamin D receptor; FoxO1, forkhead box protein O1; IGF1R, insulin-like growth factor 1 receptor; MIF, macrophage migration inhibitory factor; Calcr, calcitonin receptor; NA, not applicable.

## Conclusion

**FIGURE 3:**
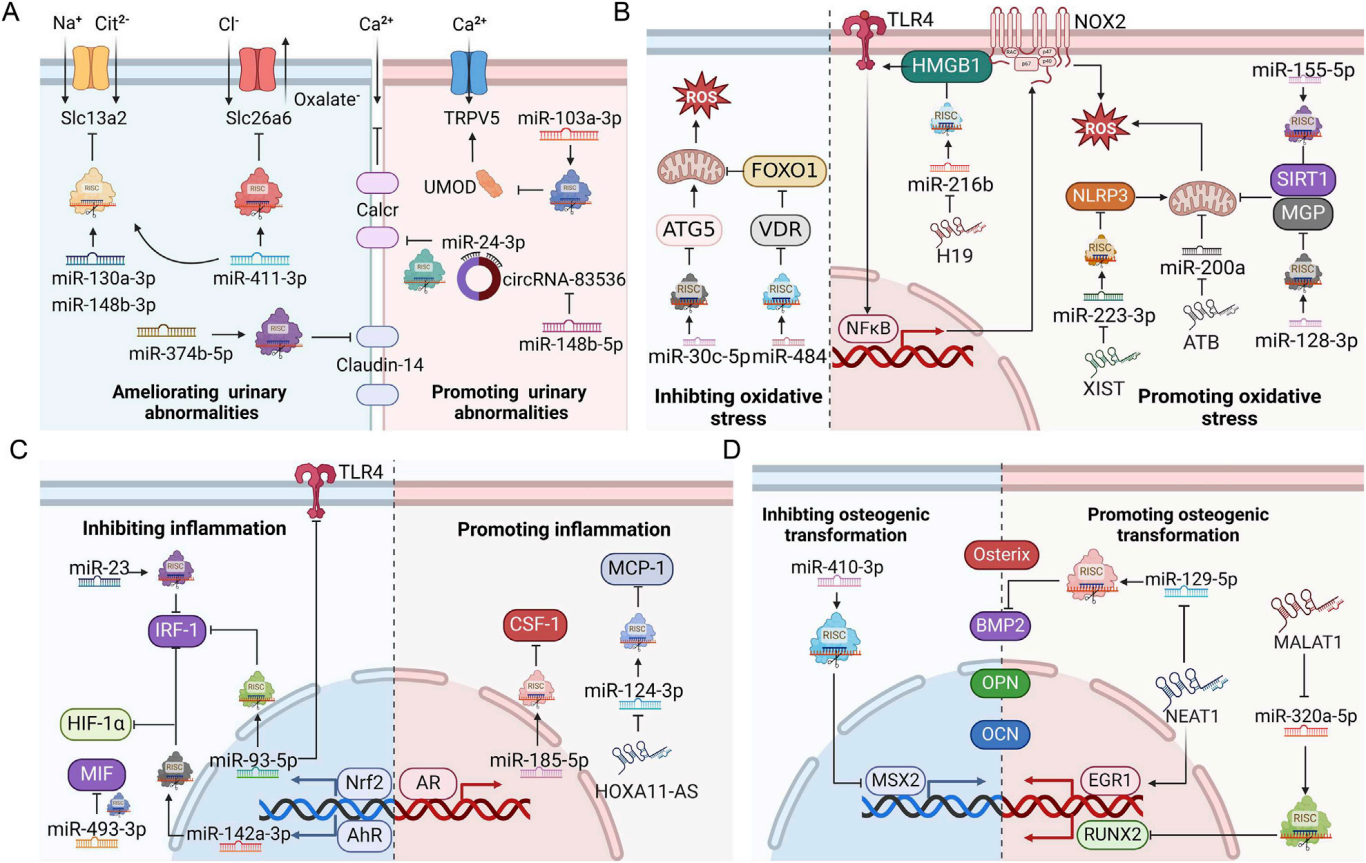
ncRNAs regulate ion transportation, oxidative stress injury, inflammation, and osteogenic transformation during stone formation. **(A)** Roles of ncRNAs in urinary ion transportation. **(B)** Roles of ncRNAs in oxidative stress. **(C)** Roles of ncRNAs in inflammation. **(D)** Roles of ncRNAs in osteogenic transformation. AhR, aryl hydrocarbon receptor; AR, androgen receptor; BMP2, bone morphogenetic protein 2; CSF-1, macrophage colony-stimulating factor 1; EGR1, early growth response protein 1; HIF-1α, Hypoxia-inducible factor 1-α; HMGB1, high mobility group protein B1; IRF1, interferon regulatory factor-1; MCP-1, monocyte chemotactic protein 1; MGP, matrix Gla protein; MSX2, homeobox protein MSX-2; NLRP3, NACHT, LRR and PYD domain-containing protein 3; Nrf2, nuclear factor erythroid 2-related 2; OCN, osteocalcin; OPN, osteopontin; RISC, RNA-induced silencing complex; ROS, reactive oxygen species; RUNX2, runt-related transcription factor 2; SIRT1, NAD-dependent protein deacetylase sirtuin-1; Slc13a2, solute carrier family 13 member 2; Slc26a6, solute carrier family 26 member 6; TLR4, toll-like receptor 4; ATG5, autophagy protein 5; TRPV5, transient receptor potential cation channel subfamily V member 5; UMOD, uromodulin; VDR, vitamin D receptor; FoxO1, forkhead box protein O1; MIF, macrophage migration inhibitory factor; Calcr, calcitonin receptor. This figure was created with BioRender.com.

The role of ncRNAs in kidney stone disease has attracted much attention in recent years, offering novel perspectives to the pathogenesis of nephrolithiasis. NcRNAs can influence stone formation by regulating ion transportation, oxidative stress, inflammation, osteoblastic transformation, autophagy, and pyroptosis (Figure 3). Among the myriad dysregulated ncRNAs, we found that miR-155 was the focus of three different studies (Jiang et al., 2020; Hu et al., 2014; Chen et al., 2020). MiR-155 is encoded by the miR-155 host gene (*mir155 hg*), which produces miR-155-3p and miR-155-5p forms. MiR-155 is strongly implicated in inflammatory autoimmune diseases (Xu et al., 2022; Zingale et al., 2021; Maciak et al., 2021). Drugs targeting miR-155 have been designed to treat T cell cutaneous lymphoma and amyotrophic lateral sclerosis in preclinical trials (Chakraborty et al., 2020). In terms of kidney stone disease, *in vitro* and animal studies suggest that miR-155 plays a lithogenic role by promoting CaOx-induced renal injury (Jiang et al., 2020; Chen et al., 2020). The expression of miR-155 is upregulated not only in renal tubular epithelial cells cultured with CaOx but also in the renal tissue, serum, and urine from kidney stone patients (Jiang et al., 2020; Hu et al., 2014; Chen et al., 2020). Therefore, can miR-155 serve as a reliable diagnostic and therapeutic target for nephrolithiasis? Well-designed prospective studies with an adequate number of kidney stone patients are expected to answer this question.

## Perspectives

Developing diagnostic and therapeutic methods related to ncRNAs holds great promise for precision medicine and personalized healthcare. Commonly employed strategies include identifying differential ncRNAs for early detection and monitoring of diseases, synthetizing antisense oligonucleotides to specifically inhibit the function of ncRNAs, developing effective delivery systems to enhance the bioavailability of ncRNAs, constructing small molecules to target specific ncRNA-protein interactions or ncRNA secondary structures, and using CRISPR-Cas9 system to precisely edit the genomic sequences encoding ncRNAs (Nemeth et al., 2024; Winkle et al., 2021; Matsui and Corey, 2017; Chen B. et al., 2022). These methods make the clinical application of ncRNA-based diagnosis and therapeutics in urolithiasis possible.

It is important to acknowledge that the majority of existing findings concerning nephrolithiasis-related ncRNAs come from cell and animal experiments, with limited studies conducted in patient populations. Although some studies have identified differential ncRNAs between kidney stone patients and healthy populations, the sample size is often small, and the diagnostic or therapeutic value of these ncRNAs has not been further confirmed using follow-up data. Moreover, the reported ncRNAs lack specificity, as they have also been implicated in other diseases. The stability of ncRNAs associated with nephrolithiasis has also not been thoroughly discussed either. These limitations currently hinder the application of ncRNAs in the diagnosis and treatment of kidney stone disease. It is anticipated that with future advances in research, ncRNAs can be used as reliable biomarkers or therapeutic targets in kidney stone disease.

## Glossary

ADSCs: mesenchymal adipose‐derived stromal cells
AGO2: argonaute 2
AhR: aryl hydrocarbon receptor
AR: androgen receptor
ATG5: autophagy protein 5
ATG7: autophagy protein 7
BMP2: bone morphogenetic protein 2
Calcr: calcitonin receptor
CaOx: calcium oxalate
CircRNA: circular RNA
CiRNA: circular intronic RNA
CLDN14: claudin-14
COM: calcium oxalate monohydrate
CRP: c-reactive protein
CSF-1: macrophage colony-stimulating factor 1
EciRNA: exonic circRNA
EGR1: early growth response protein 1
EIciRNA: exon-intron circRNA
FoxO1: forkhead box protein O1
GEO: Gene Expression Omnibus database
GHS rats: genetic hypercalciuric stone-forming rats
GO: Gene Ontology
HIF-1α: Hypoxia-inducible factor 1-α
HMGB1: high mobility group protein B1
IGF1R: insulin-like growth factor 1 receptor
IL-1β: interleukin-1β
IL-6: interleukin-6
IRF1: interferon regulatory factor-1
KEGG: Kyoto Encyclopedia of Genes and Genomes
LncRNA: long noncoding RNA
m7G: 7-methyl guanosine
MCP-1: monocyte chemotactic protein 1
MGP: matrix Gla protein
MIF: macrophage migration inhibitory factor
MiRNA: microRNA
MSX2: homeobox protein MSX-2
MUC4: mucin-4
ncRNA: noncoding RNA
NLRP3: NACHT, LRR and PYD domain-containing protein 3
Nrf2: nuclear factor erythroid 2-related 2
NXF1: nuclear RNA export factor 1
OCN: osteocalcin
OPN: osteopontin
PCR: polymerase chain reaction
PI3K: Phosphatidylinositol-3-hydroxykinase
piRNA: piwi-associated RNA
PKNOX1: homeobox protein PKNOX1
PPARA: peroxisome proliferator-activated receptor alpha
RBP: RNA binding protein
Rheb: GTP-binding protein Rheb
RISC: RNA-induced silencing complex
ROS: reactive oxygen species
rRNA: ribosomal RNA
Runx2: runt-related transcription factor 2
SD rats: Sprague-Dawley rats
SIRT1: NAD-dependent protein deacetylase sirtuin-1
Slc13a2: solute carrier family 13 member 2
Slc26a6: solute carrier family 26 member 6
snoRNA: small nucleolar RNA
snRNA: small nuclear RNA
TLR4: toll-like receptor 4
TNF-α: tumor necrosis factor-α
TNRC6: trinucleotide repeat containing adaptor 6
tRNA: transfer RNA
TRPV5: transient receptor potential cation channel subfamily V member 5
UMOD: uromodulin
UTR: untranslated region
VDR: vitamin D receptor
